# The burden of left ventricular assist device (LVAD) infections on costs, lengths of stay, antimicrobial consumption and resistance: a prospective case control approach

**DOI:** 10.1186/s13756-024-01503-4

**Published:** 2024-12-18

**Authors:** Matthew Ficinski, Jennifer West, Shannon Glassman, Katrina Wojciechowski, Jennifer Gutowski, Maryrose Laguio-Vila, Scott Feitell, Emil Lesho

**Affiliations:** 1 Infectious Diseases Department, Rochester Regional Health, 1425 Portland Avenue, Rochester, NY 14621 USA; 2 Infection Prevention, Rochester Regional Health, Rochester, NY USA; 3 Accounting Department, Rochester Regional Health, Rochester, NY USA; 4 Medical Department, Rochester Regional Health, Rochester, NY USA; 5 Cardiology Department, Rochester Regional Health, Rochester, NY USA

**Keywords:** LVAD, Cost, Length of stay, Antibiotic stewardship, Burden

## Abstract

**Background:**

Congestive heart failure has reached pandemic levels, and left-ventricular assist devices (LVAD) are increasingly used to treat refractory heart failure. Infection is a leading complication of LVADs. Despite numerous reports (most being retrospective), several knowledge gaps pertaining to the epidemiology and burden of an LVAD-associated infection (LVADi) remain. We sought to address these gaps using a prospective, case-control design.

**Methods:**

All patients who received an LVAD from November 1, 2018 to August 31, 2023 (*n* = 110) were included and prospectively monitored until death. Data were extracted from clinical encounters and medical records in real-time or near real-time and imported to Excel and REDcap electronic data capture tools. An LVADi was ascertained using definitions from the mechanical circulatory support academic research consortium in conjunction with and the U.S. National Health Safety Network. All meeting those definitions were included as ‘cases.’ Patients with no LVADi were controls. Excess lengths-of-stays (LOS) and direct costs were calculated from billing records using a commercial cost accounting software platform (Strata^®^, Chicago, IL).

**Results:**

The amount of healthcare contact before implantation and discharge to a rehabilitation or skilled nursing facility instead of home were the primary risks for infection, resulting in mean excesses of 25 hospital and 60 antibiotic-days and $43,000 per event. One-third occurred > 1 year after implantation. 35% developed > 1 infection. Gram-negative, fungal, and antimicrobial-resistant organisms predominated deep or repeat infections. 7.2% developed ≥ 3 infections. Organisms became increasingly antimicrobial resistant with subsequent infections, leading to extensive or pan-drug resistance in 4.5% of patients. The burden of an LVADi was 1862 excess hospital days, 3960 excess antibiotic days, and $3.4 million.

**Conclusions:**

Patients with LVADis had significant increases in costs, LOS, readmissions, and antibiotic usage. Antimicrobial resistance varied directly with the number of repeat infections and antibiotic exposure. Identification of factors associated with LVADi, and quantification of the burden of LVADi can inform prevention efforts and lead to reduced infection rates. As preventing infections in the first place is also important for limiting the emergence of antimicrobial resistance, we offer strategies to avoid LVADis.

**Trial registry:**

Not applicable.

## Background

Congestive heart failure has reached pandemic levels, and the prevalence in the U.S. is expected to increase by 50% over the next decade [1]. Left-ventricular assist devices (LVAD) are increasingly used to treat advanced heart failure refractory to other medical therapies. In fact, the highest number of annual implants ever reported in the history of the Interagency Registry for Mechanical Assisted Circulatory Support (INTERMACS) was recently documented [2]. 

Infection is a leading complication of LVAD therapy [1], and the epidemiology of an LVAD-associated infection (LVADi) is important for optimizing preventive strategies. Although the main registries, such as INTERMACS, publish annual quality reports, infectious and microbiologic details are often not provided [2–5]. Recent reports including such details are retrospective designs. Unlike other major surgical procedures such as coronary artery bypass grafting and total joint arthroplasty, hospitals are not required to report LVADI rates to state or federal agencies. Therefore, hospital specific reports are scarce and substantial inter hospital variability exists for infection rates [6]. As a result, knowledge gaps persist, and there have been calls for more details pertaining to duration and types of antibiotics used, species-specific culture data, and presence of implanted cardiac devices and non-LVADis [1, 3, 5, 7]. 

Therefore, we sought to address some of these epidemiologic gaps and ascertain the effect of LVADis on costs and lengths of stay (LOS) in our healthcare system, where LVADs are implanted for destination therapy only. The primary endpoints were first and subsequent LVADis, and/or death. Other outcome measures included total direct costs, and LOS. These outcomes could then be used to inform prevention efforts and bolster motivation to reduce infections by highlighting the burden of LVADi [8].

## Methods

All patients who received an implant from program inception (November 1, 2018) to August 31, 2023 (*n* = 110) were included and prospectively monitored until death. A multi-component implementation strategy consisted of the following. Data were extracted from clinical encounters and medical records in real-time or near real-time and imported to Excel and REDcap electronic data capture tools. An LVADI was ascertained using definitions from the mechanical circulatory support academic research consortium in conjunction with updated INTERMACS Appendix 3 [3–7], the U.S. National Health Safety Network [8], and the 2024 consensus statement from the International Society for Heart and Lung Transplantation [9] An LVADI was also identified when any provider caring for the patient diagnosed an LVADI and prescribed antibiotics, even if it did not fully conform to the surveillance definitions. Data were validated by independent chart review and discordant assessments were adjudicated by consensus opinion.

A case-control approach was also used, with all non-infected LVAD patients as the ‘control’ or comparator group. Excess LOS and direct costs were calculated from census data and billing records using a commercial healthcare accounting platform (Strata^®^; Chicago, IL). Total billing costs of the uninfected control patient was subtracted from the total billing cost of the infected counterpart. Antibiotic days of therapy (DOT) were defined according to CDC-NHSN guidelines. Unjustified DOT were defined as DOT not supported by culture or other microbiologic data and infection as defined by the Mechanical Circulatory Support Academic Research Consortium [3], minus 3 days allowed for empiric treatment for each unexplained fever [10]. 

A subsequent LVADI following the incident infection in the same patient was counted as an additional infection if it met any of the following criteria: (1) occurred at a different anatomic site (driveline vs. blood stream/endocarditis vs. pocket/pump vs. surgical incision, mediastinitis) than the index infection regardless of organism species or timeframe; (2) occurred > 30 days after the index infection at the same anatomic site, regardless of organism species. For same site infections requiring long-term antibiotics (i.e., endocarditis, sternal osteomyelitis), a subsequent infection was counted if it occurred any time after the full treatment course and antibiotics had been completed.

Descriptive statistics were performed on the control and infected groups using T, Chi Squared, and Mann-Whitney U tests.

## Results

The median follow-up period was 1132 days (IQR 492–1395). Sixty-seven (61%) patients developed an infection, resulting in an incidence rate of 21 infections per 100 patient-years. Thirty-five (52%), 24 (36%), and 8 (12%) of these first infections were percutaneous lead (driveline), bacteremia/endocarditis, and surgical site infections (SSI), respectively. No baseline patient characteristic, including immune status, BMI, co-morbidity, and guideline congruent perioperative prophylaxis, was associated with infection. However, the presence of a mood disorder and female gender trended towards significance. The presence of a balloon pump trended toward being negatively associated with infection (*p* = 0.06) (Table 1). The number of hospital admissions and time spent in the hospital during the year before the implant, and discharge to a facility other than home were the only baseline characteristics significantly associated with the incident infection (Table 1).

**Table 1:** Baseline patient characteristics, pre-implant hospitalizations, and disposition

	NON infected	Infected	*P*
Total	43	67	
Sex % FEMALE	18.6	34.3	0.06
Age (Mean, SD, IQR)	60.6, 12.4, 17	62.9, 10.4, 13	0.29
Race			
Caucasian%	72.1	67.2	0.59
Black%	16.3	19.4	0.68
Hispanic%	0	1.5	0.42
Asian%	2.3	1.5	0.76
Native American%	0	3	0.25
Other Race%	4.7	7.5	0.56
Unknown/Refused%	4.7	0	0.07
Ethnicity			
Non-Hispanic%	86	88.1	0.75
Cuban%	0	1.5	0.42
Puerto Rican%	2.3	4.5	0.54
Spanish%	2.3	1.5	0.76.
Unknown/Refused%	9.3	4.5	0.32
Mood Disorder %	16.3	31.3	0.08
INTERMACS (Mean, SD)	2.2, 1.0	2.4, 0.9	0.27
BMI (Mean, SD)	28.5, 6.9	30.5, 6.6	0.13
Renal Failure (%)	30.2	40.3	0.28
DM %	55.8	53.7	0.83
Immunocompromise %	9.3	7.5	0.74
Balloon Pump %	65.1	46.3	0.06
Impella %	23.3	22.4	0.91
Smoker %	19.0	27.3	0.33
Pacemaker%	71.4	65.7	0.53
No. Admissions prior to Implant	56	135	
Mean LOS/admission (SD)	6.8 d (4.9)	8.9 d (10.2)	
Total days	381	1,202	
Mean Total LOS/patient (SD)	8.9 d 4*.9*	17.9 d 10*.2*	*0.0001*
Mean Initial Implant Adm. Duration (SD)	33.6 d (27.1)	38.1 d (36.1)	0.49
Guideline Congruent Abx. Prophylaxis (%)	77.0	89.1	0.17
Disposition post implant			
Rehabilitation facility	18 (41%)	46 (69%)	0.004
Home	17 (40%)	17 (25%)	0.09
Died in hospital	8 (19%)	4 (6%)	0.03

* Immunocompromised was defined as those patients whose immune mechanisms are deficient because of immunologic disorders (e.g., human immunodeficiency virus [HIV] infection, congenital immune deficiency syndrome, cancer not in remission, or immunosuppressive therapy (e.g., radiation, cytotoxic chemotherapy, anti-rejection medication, or prednisone dose equivalent to ≥ 2 mg/kg of body weight or ≥ 20 mg/day administered for ≥ 14 consecutive days. If a patient had any of these, they were classified as immunosuppressed

48%, 10%, and 42% of infections occurred < 90, 90–120, and > 120 days from implant, respectively. There was no association with surgeon, surgical team, or operating room (Table 2). For all types of infections, the median time to first infection was 97.5 days (IQR 37–365). SSI were the earliest to develop, while percutaneous lead infections were the latest, with median times to onset of 15.5 and 200 days, respectively.

*Staphylococcus aureus* and other Gram-positive organisms were more common in superficial infections while *Pseudomonas aeruginosa* and other Gram-negatives were the predominant organisms in deep infections (Table 2). 50% of superficial infections were not cultured.

Median DOT for these first infections ranged from 10 days for superficial percutaneous lead to 79 days for deep or complicated percutaneous lead infection (Table 2).

**Table 2:** Onset of infection, organism, days of antibiotics used

Median time to infection Days	First Infection *n* = 67 [IQR]	Predominant Organisms	Antibiotic DOT Total, median, [IQR]		Second Infection *n* = 38 [IQR]	Predominant Organisms	Antibiotic DOT Total, median, [IQR]	Total	Mean total /Person
All infections 67	97.5 [37–365]		2063; 16.5 [10–42]	All 38	158.5 [37–257]		1897 37.5 [14-66.5]	3960	59.1
Uncomplicated percutaneous lead (PL) ( i.e. superficial PL) 31	142 [40–404]	No CX: 16 MSSA 9/15 CoNS 3/15 Coryn 2/15 All others 1/15 each*	932 10 [8.5–28.5]	Superficial 20	68.5 [32–224]	No Culture 6 MSSA 3/14 MRSA 2/14 *Coryn*. 1 *Staph* sp. 1 *Strep* 1 Gram negs 4	1031 17 [10–60]		
Complicated percutaneous lead (deep PL, excluding bloodstream infection) 4	200 [142–390]	No growth 1/4 Poly-microbial 2/4 *P. aeruginosa* 3/4	227 79 [27-160.5]	Deep 3	156 [171–206]	*S. epidermatus* *S. pyogenese* *P. aeruginosa* Coryne MSSA	72 47.2 mean		
Bloodstream infection 24	64 [41–434]	Streptococcal spp. 5/24 CoNS 4/24 *E. fecalis* 3/24 MSSA 2/24 MRSA 2/24 *K. pneumonia* 2/24 All others** 1/24	737 30 [15–51]	Blood contacting surface 7	214 [56–303]	*C. glabrata* 2 *Weisella confusa* 1 *P. aeruginosa* 1 MRSA 1 *E. coli* MSSA	627 49 [19–100]		
Surgical site infection 8	15.5 [9.5–46.5]	No CX 2 No growth 1/6 All other*** 1/6	167 14 [11.5–46]	SSI 2	174 [39-648.5]	No cultures 2/2	167 71 [14–83]		

External surface of implanted component (pump / pump pocket) CONS = 0 infections

*Methicillin resistant Staphylococcus aureus, Providencia rettgeri, Arcanobacterium hemolyticum, Enterococcus faecalis, Klebsiella pneumonia

** Streptococcus anginosus, methicillin resistant Staphylococcus aureus, actinomyces, ESBL E. coli, Streptococcus pneumoniae, Streptococcus parasanguinous, Streptococcus parassangunosus Streptococcus constellatus, Pseudomonas aeruginosa, corynebacterium micrococcus Streptococcus viridans, micrococcus

*** Staphylococcus epidermidis, Klebsiella Pneumo Candida glabrata, Streptococcus dysgalactiae, Streptococcus mitis oralis, Streptococcus para sanguinous, bacillus species, Peptostreptococus

CX = culture; CONS = Coagulase negative Staphylococcus species; Coryne = Corynebacterium; MSSA Methicillin Sensitive Staphylococcus aureus; MRSA Methicillin Resistant Staphylococcus aureus

The most commonly used antibiotics were vancomycin and cefazolin. Device driveline trauma was significantly associated with infection (Table 3). Infected patients required significantly more 30- and 90-day readmissions for any reason (including infection), and longer LOS than uninfected (Table 3). Neurologic dysfunction with central nervous system injury and major bleeding were not significantly different between the infected and uninfected groups. The time from device implantation to death was shorter in the uninfected group, trending towards but not reaching significance (Table 3).

**Table 3:** Post left ventricular device implant events, costs, and antibiotic consumption

	Uninfected *N* = 43	Infected *N* = 67	*P*	Excess
Device Trauma (%)	16.3	40.3	0.008	
Mean no. 30d readmissions / patient (SD)	1.4 (0.7)	2.4 (1.7)	0.0004	
Mean no. 90d readmissions/patient (SD)	1.5 (0.9)	3.3 (2.5)	0.0001	
Total No. post implant readmissions	50	231		
Mean LOS/admission (SD)	7.1 (9.4)	9.6 (12.9)	0.19	2.5 days
Total days / study period	355	2,218		1863
Mean Total days/person	8.3 *(9.4)*	33.1 *(12.9*)	0.0001	24.8
Driveline relocation or explanation	0	8 (12%)		
Exploration of chest cavity/mediastinum	2	2	0.64	
Neurologic Dysfunction w CNS Injury	4 (8%)	9 (13%)	0.41	
Major Bleeding	8 (19%)	16 (24%)	0.53	
Mortality	10 (23%)	9 (13%)	0.17	
Median days from implant to death (IQR)	38.5 (26–662)	616 (179–889)	0.06	
Non-LVAD infection	23	42	0.30	
Direct Costs				
Labor	$11,745	$12,551		
Supplies, Pharmacy, Other	$5,274	$5,640		
Total /admission	$17.019	$18,191		$1,172
Total for study period	$850,950	$4,202,121		$3,351,171
Mean total / person	$19,790	$62,718		$42,928
Antibiotic Consumption				
	No infection of any type (*n* = 20)	Non-LVAD Infection	LVAD infect.	
Total Days of Therapy^	0	253	3960	4,213
Mean unjustified Days^	0	3	13	

**Fig. 1:**
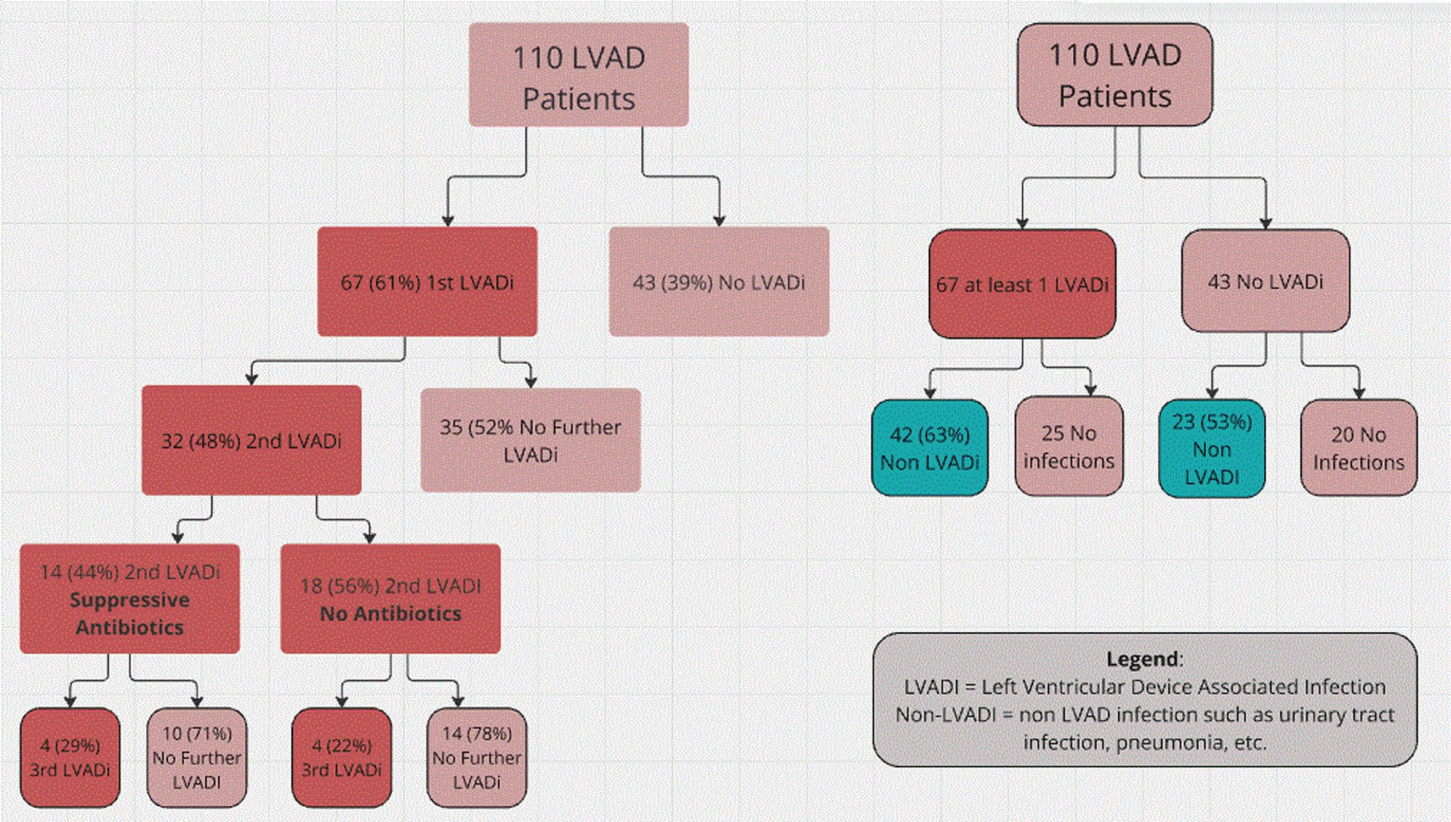
FLow Diagrams of Patients Who Received Left Ventricular Assist Devices

Thirty-two patients (48%) developed a second device-associated infection (Table 2). Median time to the second infection was 159 days (IQR: 37–257). Compared to the first infection, there were more Gram-negative organisms (Pseudomonas and enterics, and fungal organisms including *Candida glabrata* and *Candida auris*) associated with subsequent infections. Eight (7.2%) developed three or more infections. Fourteen (44%) of the patients with a second LVAD infection received long term suppressive antibiotics. Four of those (29%) developed a third infection (Fig. 1). Eighteen (56%) of patients with a second LVAD infection were not placed on suppressive antibiotics, and four (22%) developed a third LVAD infection (Fig. 1). Organisms became increasingly antibiotic-resistant with subsequent infections progressing in some cases to extensive or pan resistance. One such patient was successfully treated with anti-staphylococcal and anti-pseudomonas phage therapy in combination with antibacterials [11]. 

23 (53%) of patients who did not develop any LVAD infection had a non-LVAD associated infection, while 42 (63%) of patients with an LVAD infection also developed non-LVAD associated infection (Fig. 1). The most common non-LVADi was pneumonia followed by sepsis (Table 4). These non-LVADis were more common in the group with an LVADi.

**Table 4:** Non-LVAD associated infections

	No LVAD Infection	LVAD Infection
Patients Non LVAD Infections	23 patients 23/43 = 53%	42 patients 42/67 = 63%
Number of Non LVAD infections	30	46
PNA/LRTI	10 (33%)	16 (35%)
Sepsis / SIRS	7 (23%)	6 (13%)
GI/enteritis	4 (13%)	6 (13%)
Pacer ICD	2 (7%)	0
UTI	2 (7%)	6 (13%)
Cellulitis	2 (7%)	5 (11%)
All others combined	3 (10%)	7 (15%)

The excess cost and LOS associated with an LVADi was $43,000 and 25 days per patient per event. Non LVAD infections can also increase costs and lengths of stay. Since there are so many different types of non-LVAD infections (i.e., *C. difficile*, uncomplicated or complicated UTI, pneumonia of varying severity and distribution, cellulitis, SIRS / sepsis, etc.) we are unable to state a general overall cost for non-LVAD infections. However, we do know the costs of certain specific healthcare associated infections (HAI) during the same timeframe at this facility. Per admission, the composite mean excess cost for HAI such as CLABSI, CAUTI, and SSI was $34,298 with 9.3 excess days of stay [12]. 

Overall, an LVADi also resulted in a mean of 60 antibiotic DOT. The median unjustified DOT was 13 (IQR 3–20). Mortality rates were not significantly different between patients with an LVADi and those without an infection, but the latter had shorter survival times post-implant. LVADs were present for a median of 38.5 days (IQR 26,662) at time of death in the uninfected group versus 616 days in the group with LVADi. This difference trended towards but did not reach significance (Table 3).

## Discussion

LVADi led to significant increases in costs and hospital LOS. Over this 5-year observation of 110 LVAD patients, the total burden of device-related infections was 1862 excess hospital days, 3960 excess antibiotic days, and $3.4 million. Infected patients had triple the number of re-admissions and twice the number of hospital days. These values likely underestimate the true burden of infection because the excess costs and LOS of each subsequent infection in the same patient could not be fully captured.

Two baseline characteristics or risks contributed to LVADi. The first was the level of healthcare exposure during the year prior to implantation (Table 1). As healthcare contact has been shown to be associated with increased colonization with important pathogens, this finding is biologically plausible [13]. 95% of admitting diagnoses prior to the implantation were cardiac related such as decompensated heart failure, dysrhythmia, implantable cardiac device insertion, and cardiac catheterization. 2% were for acute sever kidney failure or dialysis, and all other diagnoses (i.e., orthopedic, infection, gastrointestinal etc.) accounted for 3% of pre-implantation admissions. The second risk for LVADi was being discharged to a skilled nursing or rehabilitation facility after the implantation admission. Patients discharged to facilities other than home may receive less close attention if there are staffing shortages. Notably, 34% of LVADi occurred over a year after the implantation, hinting at potential influences from patient or socioeconomic factors, rather than management or device-related factors. Such factors included supply shortages (i.e., chlorhexidine), diminution or loss of financial and/or social support, and substance abuse.

The shift to Gram-negative and fungal organisms in repeat infections was likely due to selection pressure from prior antibiotics. More patients in the non-infected group had intra-aortic balloon pumps (IABPs), and the presence of an IABP trended towards a statistical negative association, appearing to have a preventive influence (Table 1). This could be explained by the fact that these patients had more chlorhexidine (CHG) exposure via daily bathing and device scrubbing with CHG wipes.

Our findings related to timing, frequency, type of infection, microbiology, and a relative lack of baseline patients’ characteristics associated with infection are similar to other studies [2, 7, 14]. However, we did not find younger age or BMI to be associated with a higher risk of infection. This might be due to the fact that the mean BMI in the uninfected group was also borderline obese and not significantly lower than the infected group. It could also be because other unknown characteristics or variables canceled the effect of BMI on infection risk. The time of death in the non-LVADi group was shorter than the LVADi group most likely because more patients in in the non-LVADi group died from early noninfectious post-operative complications before being discharged from the implant admission (Table 1). The percentage of mortality was 23% in the uninfected group and 13% in the group with an LVAD. Although statistically not significant, this is likely due to the fact that the people in the noninfected group had higher rates of early severe complications such as end-stage multi-organ failure and more of those patients were placed on comfort care / withdraw/ of support by their families.

Our study is notable for including more baseline characteristics (such as mood disorder, the presence of other intra-cardiac devices, and balloon pumps) than prior studies, as well as the type and duration of antibiotics. There are conflicting data regarding the role of diabetes, renal disease, and history of depression in subsequent LVAD infection. We did not find any of those characteristics to be associated with infection. However, the presence of an intra-aortic balloon pump (IABP) trended towards having a negative association with infection. We speculate that finding might be due to closer and more frequent nursing attention, along with increased and regular use of topical disinfectants such as chlorhexidine (CHG) wipes). We observed that CHG use was not consistently applied to ICU patients without balloon pumps, averaging only 60% of the time. Also, whenever possible, axillary approaches were used for IABP placement to reduce a patient’s bed-bound status.

Another strength distinguishing our work from prior studies is the prospective nature involving real-time manual review of every patient’s clinical encounters. Furthermore, by identifying factors associated with LVADi and highlighting the burden of LVADi to all stakeholders, significant reductions in other cardiac surgical infections were observed [15]. 

This was achieved by increasing pre- implant or procedural patient education and continual education throughout life of the device, using surgical anchors and VAD specific gowns for inpatients or shirts/vests for outpatients to decrease driveline trauma. We also intensified pre and post procedural bathing protocols to include CHG bathing for at least 6 weeks post op, then every 3 days for the life of the device. We educated providers and nurses on the importance of CHG bathing for all readmissions even when fully healed, and the importance of dressing continuity and disposable EKG leads.

Limitations of this report include being from a single center, and only the hospitals and payers’ perspective were considered. To account for immortal time bias we collected data on the exact time of exposure initiation (implant date) and the time of event occurrence (date of infection), ensuring the time origin was the same for both groups.

Despite limitations, this observation provides insight into some knowledge gaps regarding the microbiology, antibiotic consumption, and the burden associated with infections in LVAD patients. These findings highlight the importance of minimizing avoidable healthcare contact and delays before LVAD implantation once a patients’ heart failure has become refractory to all other treatments. Our findings also underscore the value of fostering outpatient and community-based social support mechanisms for patients after discharge.

## Data Availability

No datasets were generated or analysed during the current study.
